# Editorial: New Insights Into B Cell Subsets in Health and Disease

**DOI:** 10.3389/fimmu.2022.854889

**Published:** 2022-02-01

**Authors:** Veerle Somers, Deborah K. Dunn-Walters, Mirjam van der Burg, Judith Fraussen

**Affiliations:** ^1^University MS Center (UMSC), Hasselt-Pelt, Belgium; ^2^Department of Immunology and Infection, Biomedical Research Institute, Hasselt University, Hasselt, Belgium; ^3^School of Biosciences and Medicine, University of Surrey, Guildford, United Kingdom; ^4^Laboratory for Pediatric Immunology, Department of Pediatrics, Willem-Alexander Children’s Hospital, Leiden University Medical Center, Leiden, Netherlands

**Keywords:** B cell subsets, B cell function, B cell metabolism, B cell vaccination response, B cell biology, B cell pathology

B cells play a pivotal role in both humoral and cell-mediated immune responses. The B cell population is made up of different B cell subsets depending on their developmental or proliferation status, location and function. B cells produce “natural” antibodies as part of innate immunity, but also generate antigen-specific immunity during adaptive immune responses. B cell development starts in the bone marrow from hematopoietic stem cells. Precursor B cells then enter the circulation as transitional B cells and further mature into naive B cells. After antigen recognition, naive B cells become activated and either develop into short-lived plasma cells secreting antigen-specific antibody, or enter a germinal center (GC) reaction where antigen binding is further optimized by hypermutation of the immunoglobulin genes and affinity maturation. The GC reaction occurs in secondary lymphoid tissue and leads to the generation of high affinity, long-lived, antibody-secreting and memory B cells. Antibody effector functions can be modified by isotype switching.

An increasing number of different B cell subsets have been described in recent years in relation to different pathologies such as autoimmunity, immune deficiencies and cancer. Innovative technologies are now available that enable in-depth studies in B cells. This has resulted in a complex maze of human B cell subsets with different identifying markers and functions. Therefore, the goal of this Research Topic was to highlight these recent exciting studies of B cell subsets and their role in health and disease. The contributions received for this Research Topic consisted of high-quality research papers and detailed overview papers that covered four main areas: (1) newly-identified B cell subsets, (2) mechanisms of B cell biology and pathology, (3) vaccination response and (4) B cell metabolism.

## Newly-Identified B Cell Subsets

Studies analyzing novel B cell subsets using deep phenotyping and single-cell transcriptomics are an important section of this Research Topic. Unraveling the phenotype and function of different B cell subsets is crucial to understand complex B cell biology and its involvement in multifactorial pathologies in order to develop disease treatment strategies. In three original publications, deep phenotyping was used to study novel B cell subsets that were originally linked with aging and are now studied more widely in multiple pathologies. Wilbrink et al. reported elevated levels of a heterogeneous subset of CD27^-^CD38^lo^CD21^lo^ B cells in axial spondyloarthritis (axSpA) patients by flow cytometry that were correlated with the presence of extra-skeletal manifestations. These findings support a possible role for B cells in the pathogenesis of axSpA. Rincon-Arevalo et al. used mass cytometry-based immunophenotyping to study CD11c^+^ B cells in patients with systemic lupus erythematosus (SLE) and primary Sjögren’s syndrome (pSS). They showed that elevated CD11c^+^ B cells in SLE and SS are a heterogeneous subset with a distinct expression pattern of checkpoint molecules that could indicate their role in dysregulated immune activation in autoimmune disease. The above mentioned B cell subsets show characteristics of atypical memory B cells that are induced *via* an extrafollicular B cell activation route.

Next to this, Ruschil et al. reported on elevated levels of another atypical B cell subset, the IgD^-^CD27^-^ double negative (DN) B cells, also defined here as CD20^lo^, in several inflammatory neurologic diseases. Interestingly, DN B cells could be induced upon vaccination in healthy individuals, which induced a transient clonal expansion of DN B cells reactive against the vaccine antigen. In single-cell transcriptomic analysis, DN B cells did not show clear clustering according to their gene expression profile but clustered with naive B cells, memory B cells and plasmablasts. These observations underlined the heterogenous nature of the DN B cell subset, which was also demonstrated by another paper in this Research Topic by Stewart et al. This publication brought new insights into different subsets of DN B cells while providing a reference single-cell dataset for total circulating B cell subsets. Four different DN B cell clusters were identified that could be divided into two main developmental branches: a T-independent branch that is associated with an extrafollicular response (DN2/3) and a T-dependent branch including precursors of classical memory B cells (DN1/4). The newly described DN4 B cell subset was IgE-rich and could be linked to an allergy response in one of the included subjects.

Two studies performed a more general B cell phenotyping in relation to different pathologies. Simon et al. demonstrated a decrease of a DN B cell subset, namely IgD^-^CD27^-^CD38^+^ DN1 B cells, in systemic sclerosis (SSc) patients with active versus inactive disease while IgD^-^CD27^+^CD38^-^CD95^+^ activated switched memory B cells were increased in SSc patients with more severe disease. These B cell subset changes could reflect their involvement in disease activity and pathology. Moreover, differential B cell subset dynamics was indicated at different stages following primary HIV infection by Jiménez et al., as characterized by early B cell activation associated with antiviral responses and later changes after sustained viral infection.

Antibody secreting cells and liver B cell subsets were reviewed in 2 papers. Zografou et al. provided a detailed overview of the different characteristics of short- and long-lived antibody secreting cells and evidence for their involvement in IgG1 and IgG4 autoantibody-mediated neurological disorders. In their review, Patel et al. gathered information on liver B cell subsets and the contribution of B cells to the pathology of multiple liver diseases, and critically discussed the impact of B cell depletion therapy in the liver setting.

## Mechanisms of B Cell Biology and Pathology

Five original contributions to this Research Topic focused on mechanisms of B cell biology and pathology. Dernstedt et al. used primary human tonsillar B cells to show that the expression of the complement regulatory protein Decay Accelerating Factor (DAF) is downregulated on GC B cells in order to prime them for complement-dependent phagocytosis. Moreover, analysis of human bone marrow samples indicated that DAF is also regulated during B cell development with an upregulation in the late developmental stages. Thus, this study revealed a novel role of DAF both in GC B cell phagocytosis and B cell development. Picón et al. showed that highly inflammatory multiple sclerosis (MS), characterized by the presence of lipid-specific oligoclonal IgM bands in the cerebrospinal fluid, can counteract the effect of age in the inflammation of the adaptive immune system. This was shown by the absence of an age-related decrease in B and T cell numbers and an increase in anti-cytomegalovirus antibodies in MS patients with lipid-specific oligoclonal IgM bands compared to those without these oligoclonal IgM bands. Another MS study, by Smets et al., focused on the involvement of B cell activating factor (BAFF) in the working mechanism of fingolimod and interferon-beta treatment for MS. Both treatments induced BAFF which contributed to a shift in B cell subset composition towards transitional B cells although B cell regulatory cytokines, known to be increased in transitional B cells, were not upregulated. These findings shed more light onto the mechanisms behind the failure of BAFF-depleting strategies in MS treatment. In this regard, Wiedemann et al. further elucidated the involvement of another important B cell related molecule, the inhibitory checkpoint molecule B- and T-lymphocyte attenuator (BTLA), in SLE pathology. SLE B cells presented with reduced BTLA expression and lack of inhibition during B cell differentiation into memory B cells and plasmablasts, suggesting an intrinsically abnormal checkpoint function of BTLA. However, inhibition of a key downstream phosphokinase, SYK, mimicked the effects of BTLA activity *in vitro* and could thus potentially overcome these B cell disturbances in SLE. Another original contribution by Su et al. used high-throughput sequencing of the Ig heavy chain repertoire to study B cell involvement in membranous nephropathy (MN), an autoimmune glomerular disease. MN patients presented with abnormalities in CDR3 length, hydrophobicity, somatic hypermutation and germ line index. Importantly, several Ig heavy chain characteristics, including the usage of specific transcripts, CDR3 length and somatic hypermutation rate, could predict therapy efficacy, which points to the potential use of Ig heavy chain repertoires as theranostic biomarker for MN.

## Vaccination Response

Vaccination-induced changes in B cell subsets and responses were addressed in two contributions to this Research Topic. Tjiam et al. examined the antigen-specific B cell response to tetanus toxoid (TTd) booster vaccination using dual-TTd tetramer flow cytometry in combination with unsupervised analysis methods. The antigen-specific B cell response to vaccination was highly dynamic, showing pre-vaccination antigen specificity in IgM^+^CD27^+^ B cells followed by expansions of IgG^+^ plasmablasts at 7 days, and IgG^+^ memory B cells at 14 days, after vaccination. Moreover, the observed increase in TTd tetramer binding of IgG^+^ memory B cells post-vaccination could be predicted by frequencies of activated (PD-1^+^ICOS^+^) circulating follicular helper T cells. The applied workflow has potential to be used for evaluation of vaccination outcome, a subject which was thoroughly discussed by Diks et al. This systematic review provided a detailed overview of factors influencing vaccine responsiveness in individuals with a compromised immune system, including the elderly, patients with a primary or secondary (HIV) immunodeficiency, splenectomised individuals and individuals under immunosuppressive treatment. The most significant predictors of vaccine efficacy were reduced memory B cell numbers, the presence of atypical B cell subsets (exhausted/activated) and pre-existing immunological memory.

## B Cell Metabolism

B cell metabolism was discussed in two publications. Metabolic pathways regulating B cells were extensively reviewed by Iperi et al. with a focus on regulatory B cells, oncogenic and autoimmune B cell processes. They highlighted the differential involvement of different metabolic pathways depending on B cell maturation, functional activities, stimulatory context and microenvironment. Huang et al. studied B cell metabolism in the context of decompensated hepatitis B virus (HBV)-related liver cirrhosis (D-LC), a condition that results in an increased occurrence of infections and reduced vaccination efficacy. They showed that T-dependent B cell responses were impaired in HBV D-LC patients, possibly due to dysfunctional energy metabolism. *In vitro* activated B cells from the HBV D-LC patients demonstrated reductions in OXPHOS and glycolysis which could cause the observed B cell hyporesponsiveness.

## Conclusion

In conclusion, this Research Topic provides an overview of the involvement of different B cell subsets in normal B cell biology and in multiple pathologies ranging from autoimmune conditions to infectious diseases/vaccination, immunodeficiency and liver pathology. Continued analysis of the complex heterogeneous nature of B cell subsets and function is needed to understand how to apply B cell-targeting therapeutic strategies in different clinical settings.

## Author Contributions

VS, DD-W, MB and JF edited the Research Topic and wrote this editorial. All authors approved the submitted version of this editorial.

## Conflict of Interest

The authors declare that the research was conducted in the absence of any commercial or financial relationships that could be construed as a potential conflict of interest.

## Publisher’s Note

All claims expressed in this article are solely those of the authors and do not necessarily represent those of their affiliated organizations, or those of the publisher, the editors and the reviewers. Any product that may be evaluated in this article, or claim that may be made by its manufacturer, is not guaranteed or endorsed by the publisher.

